# High-throughput SuperSAGE for gene expression analysis of *Nicotiana tabacum*–*Rhizoctonia solani* interaction

**DOI:** 10.1186/s13104-017-2934-9

**Published:** 2017-11-21

**Authors:** Roxana Portieles, María Elena Ochagavia, Eduardo Canales, Yussuan Silva, Osmani Chacón, Ingrid Hernández, Yunior López, Mayra Rodríguez, Ryohei Terauchi, Carlos Borroto, Ramón Santos, Melvin D. Bolton, Camilo Ayra-Pardo, Orlando Borrás-Hidalgo

**Affiliations:** 1 Center for Genetic Engineering and Biotechnology, 10600 Havana, Cuba; 2 Tobacco Research Institute, Carretera de Tumbadero 8, 6063, San Antonio de los Baños, Havana, Cuba; 3 Iwate Biotechnology Research Center, Kitakami, Iwate 024-0003 Japan; 4 Centro de Investigación Científica de Yucatán, Calle 43 No. 130, Colonia Chuburná de Hidalgo, 97200 Mérida, Yucatán Mexico; 5 Universidad Técnica Luis Vargas Torres de Esmeraldas, Av. Kennedy 704, Esmeraldas, Ecuador; 6 USDA-Agricultural Research Service, Northern Crops Science Laboratory, 1605 Albrecht Blvd., Fargo, ND 58102-2765 USA; 7 Henan Provincial Engineering Laboratory of Insect Bio-reactor, Nanyang Normal University, Henan, 473061 People’s Republic of China; 8 Shandong Provincial Key Laboratory of Microbial Engineering, School of Biotechnology, Qi Lu University of Technology, Jinan, 250353 People’s Republic of China

**Keywords:** *Rhizoctonia solani*, SuperSAGE, Tobacco, Salicylic acid

## Abstract

**Objective:**

The ubiquitous soil pathogen *Rhizoctonia solani* causes serious diseases in different plant species. Despite the importance of this disease, little is known regarding the molecular basis of susceptibility. SuperSAGE technology and next-generation sequencing were used to generate transcript libraries during the compatible *Nicotiana tabacum*–*R. solani* interaction. Also, we used the post-transcriptional silencing to evaluate the function of a group of important genes.

**Results:**

A total of 8960 and 8221 unique Tag sequences identified as differentially up- and down-regulated were obtained. Based on gene ontology classification, several annotated UniTags corresponded to defense response, metabolism and signal transduction. Analysis of the *N. tabacum* transcriptome during infection identified regulatory genes implicated in a number of hormone pathways. Silencing of an mRNA induced by salicylic acid reduced the susceptibility of *N. tabacum* to *R. solani*. We provide evidence that the salicylic acid pathway was involved in disease development. This is important for further development of disease management strategies caused by this pathogen.

**Electronic supplementary material:**

The online version of this article (10.1186/s13104-017-2934-9) contains supplementary material, which is available to authorized users.

## Introduction

*Rhizoctonia solani* Kühn is a soil-borne pathogen that produces disease in many agriculturally-important crops throughout the world. Although no race structure is defined for this species, isolates are grouped based on hyphal anastomosis reactions and consequently are placed into so-called anastomosis groups (AGs) [1]. In tobacco (*Nicotiana tabacum*), *R. solani* AG-2-2 and AG-3 cause damping-off, stem rot and sore shin (a plant seedlings disease characterized by stem cankers that girdle the stem near the soil line) in older plants [2, 3]. Seedling death often occurs in the greenhouse while moderate to severe stunting and/or plant death may occur in the field in plants afflicted with this disease. Transplanting infected but non-symptomatic seedlings disperses the pathogen and is a common cause of sore shin in field plants. However, AG-2-2 and AG-3 already present in a field can also initiate infection [4]. Management of this disease can be difficult using conventional means because *R. solani* can subsist for long period in soil as mycelium or sclerotia [1].

Resistance to *R. solani* in tobacco cultivars is highly desirable. However, knowledge of tobacco resistance mechanisms to *R. solani* is insufficient. Various lines of evidences suggest multiple responses involving constitutive and induced mechanisms controlled by numerous defense pathways [1]. Though the identification of resistance to *R. solani* has been successful in crops such as peanut, bean, rice, sorghum, and sugar beet [5–9], resistance screens in tobacco germplasm have been limited to a small number of commonly used cultivars and the disease remains a major economic factor for tobacco growers. Under low disease pressure, significant differences in both stem rot and target spot disease incidence could be observed among the different genotypes. However, resistance to target spot was not observed when disease pressure was high in any genotype, and partial resistance to stem rot was observed only in a few genotypes [4].

Knowledge of the molecular basis of *R. solani* susceptibility in tobacco is scarce. For example, type III knockdown tobacco lines targeting calmodulin (CaM) *NtCaM13* exhibited a strong increased in susceptibility during a compatible interaction. This suggests that type III CaM isoforms may be involved in basal defense against *R. solani* independent of jasmonic acid (JA) and ethylene (ET) signaling [10].

Here, we used SuperSAGE and next generation sequencing for the analysis of the transcriptome of *N. tabacum* after inoculation with a compatible *R. solani* strain. We studied the genes encoding potential regulatory components in *N. tabacum* plants by looking for high and low abundant transcripts that were rapidly and transiently induced after the inoculation with the pathogen. Subsequently, virus-induced gene silencing (VIGS) demonstrated the contribution of an mRNA inducible by salicylic acid (SAR8.2k) in the susceptibility of *N. tabacum* to *R. solani*.

## Main text

### Results and discussion

Two SuperSAGE libraries were generated from *N. tabacum* mock-inoculated and inoculated with *R. solani* (Additional file 1). Plant samples were harvested at several time-points during *R. solani*–*N. tabacum* interaction. The total number of SuperSAGE tags obtained was 2,838,239, comprising 1,436,124 tags from mock-inoculated plants and 1,402,115 from inoculated plants (Additional file 2: Table S1). These tags represented 649,531 unique sequences, and 11,286 detected differentially regulated with a *P* < 0.05. A total of 8960 and 8221 unique Tag sequences identified as differentially up and down expressed were obtained after inoculation with *R. solani*, respectively.

**Fig. 1:**
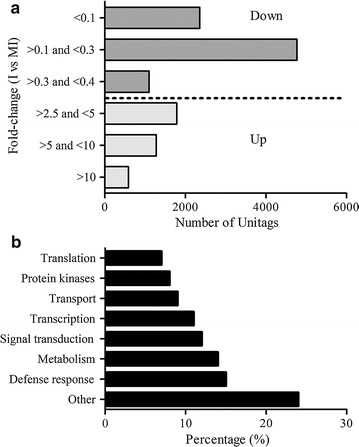
Analysis of differentially expressed UniTags. **a** Fold change [inoculated (I) vs. mock-inoculated (MI)] distribution of the differentially expressed UniTags. **b** Distribution of annotated UniTags in gene ontology (GO) categories based on molecular function and biological process

Most of the down-regulated UniTags presented fold-change (FC) values between 0.1 and 0.3, followed by up-regulated UniTags presenting FC values 2.5 and 5, respectively (Fig. 1a). Among the most prevalent gene ontology (GO) biological processes were those classified as defense response related (15%), followed by metabolism (14%), signal transduction (12%), transcription (11%), transport (9%), protein kinase (8%), translation (7%) and 24 into other functions (Fig. 1b). Finally, the top up- and down-regulated annotated tags in tobacco plants inoculated with *R. solani* are listed in Additional file 3: Table S2.

**Fig. 2:**
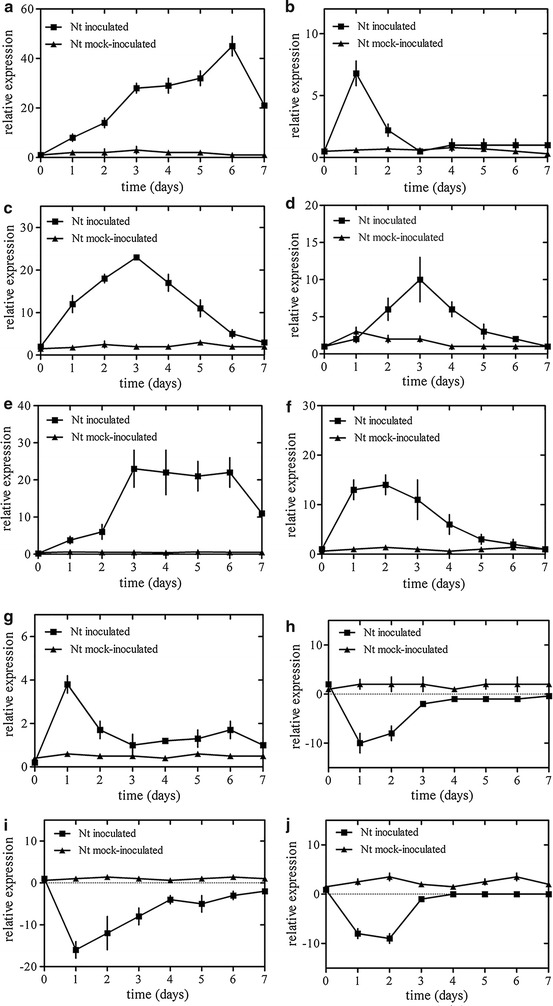
Analysis of mRNA accumulation corresponding to selected UniTags during the interaction. Transcripts levels were analyzed in three biological replicates (n = 3). Bars indicate standard error of the mean and least significant difference at P < 0.05. **a** Jasmonate ZIM-domain protein 3b; **b** receptor-like cytosolic serine/threonine-protein kinase RBK1; **c** mRNA inducible by salicylic acid (SAR8.2k); **d** DNA methyltransferase 1-associated protein 1; **e** auxin-repressed 12.5 kDa protein-like; **f** BRI1 kinase inhibitor 1-like; **g** DELLA protein GAI-like; **h** xyloglucan endotransglucosylase/hydrolase protein 15; **i** pectinesterase/pectinesterase inhibitor U1; **j** auxin-responsive protein IAA13 isoform 4X

A group of ten differentially expressed UniTags were selected to study the kinetic of mRNA induction by qPCR. Total RNA was extracted from inoculated *N. tabacum* and mock-inoculated *N. tabacum* plants at different time points after inoculation. Tobacco plants showed a quick and strong induction of receptor-like cytosolic serine/threonine-protein kinase RBK1, BRI1 kinase inhibitor 1-like and DELLA protein gene after 1 days post inoculation, respectively. Meanwhile, the expression of the xyloglucan endotransglucosylase/hydrolase protein 15 and pectinesterase/pectinesterase inhibitor U1 gene were rapidly down regulated after 1 day post-inoculation. The auxin-responsive protein IAA13 isoform X4 gene was found to be significantly silenced after 2 days post-inoculation. The expression of jasmonate ZIM-domain protein 3b gene was up-regulated after 6 days post-inoculation. Meanwhile, the expression of mRNA inducible by salicylic acid (SAR8.2k), DNA methyltransferase 1-associated protein 1 and auxin-repressed 12.5 kDa protein-like gene were induced after 3 days post-inoculation (Fig. 2).

To test whether the subset of seven differentially up expressed UniTags contributes to the disease susceptible phenotype observed in *N. tabacum*, we silenced their expression in *N. tabacum* using virus-induced gene silencing (VIGS). Infiltration with TRV-based constructs containing a *PDS* fragment resulted in extensive bleaching indicative of reduced *PDS* expression. However, this behavior was not observed in all the plants analyzed. Therefore, gene expression of all seven targeted genes was evaluated by qPCR and only those plants showing significantly lowered transcript levels were used for further evaluation (Additional file 4). VIGS of a gene inducible by salicylic acid (SAR8.2k) reduced the susceptibility to *R. solani*, suggesting the importance of this gene in the susceptibility to this necrotrophic pathogen (Table 1). Although, high resistance in the silenced plants was not observed, *N. tabacum* was clearly less affected by the pathogen in terms of percentage of damping-off. Meanwhile, other genes did not exhibit a significant contribution.

**Table 1:** Functional analysis of the up regulated gene of tobacco plants inoculated with *Rhizoctonia solani*

Genetic construction	Disease incidence Percentage of damping-off
Non-silenced *N. tabacum* cv. ‘Sumatra’	63^a^
pTV::*NtPDS*	61^a^
pTV::JZDP	64^a^
pTV::bak1	62^a^
pTV::mRNA ISA	39^b^
pTV::MTAP	63^a^
pTV::ARP	64^a^
pTV::bki1	62^a^
pTV::DP	64^a^
Standard error	5.2

Data with the same letter in each column, are not significantly different

Values are the mean of three replicates with 10 plants each. The disease reaction was evaluated 7 days post inoculation with *R. solani*. *PDS* is phytoene desaturase as a VIGS control. (JZDP) jasmonate ZIM-domain protein 3b; (bak1) receptor-like cytosolic serine/threonine-protein kinase RBK1; (mRNA ISA) mRNA inducible by salicylic acid; (MTAP) DNA methyltransferase 1-associated protein 1; (ARP) auxin-repressed 12.5 kDa protein-like; (bki1) BRI1 kinase inhibitor 1-like; (DP) DELLA protein GAI-like; (XEHP) xyloglucan endotransglucosylase/hydrolase protein 15; (PMEI) pectinesterase/pectinesterase inhibitor U1; (IAA) auxin-responsive protein IAA13 isoform X4

Salicylic acid and jasmonic acid are important factors that influence signaling networks implicated in induced defense responses against diverse pathogens [11]. Generally speaking, biotrophic pathogens tend to be more sensitive to salicylic acid dependent responses, whereas resistance to necrotrophic pathogens that benefit from cell death is typically mediated by jasmonic acid dependent defenses [12]. Additionally, it is also well-known that salicylic acid and jasmonic acid dependent pathways can be antagonistic [13, 14]. We provide evidence that the salicylic acid pathway might contributes to disease development caused by *R. solani* in tobacco. Pathogens use a myriad of forms to encourage virulence, defeat plant immune responses, and colonize their hosts [15]. Interestingly, salicylic acid showed to promote disease development caused by the necrotrophic pathogens *Botrytis cinerea* and *Alternaria solani* in *Arabidopsis* and tomato plants, respectively [16–18]. The gene inducible by salicylic acid (SAR8.2k) identified may be involved in the susceptibility response of *N. tabacum* to necrotrophic pathogen *R. solani*. Our data suggest possible strategies used by this pathogen to overcome plant defense and, subsequently, develop disease. This knowledge will be useful for the development of disease management strategies for this important pathogen.

### Methods

#### Fungal and plant materials, and infection assays

*Nicotiana tabacum* cv. ‘Sumatra’ plants were grown in pots (6-in.) containing the substrate of black turf and rice husk (4:1) and maintained in growth chambers at a temperature of 23 °C. The anastomosis group 2–2 of *R. solani* isolated from infected tobacco plants was grown and prepared for the inoculation procedure according to Shew and Main [19]. Two-week-old tobacco seedlings were inoculated with six rice infested grains from *R. solani* [4]. Also, mock-inoculated *N. tabacum* cv. ‘Sumatra’ plants were used as controls.

#### Construction of SuperSAGE libraries

Two SuperSAGE libraries were obtained to isolate and identify transcripts that were differentially induced in *N. tabacum* cv. ‘Sumatra’ plants (2-week-old) inoculated and non-inoculated with *R. solani*. Basically, the library was prepared with the following samples:

- *Control sample* Pool prepared from root, stem and leaves from *N. tabacum* cv. ‘Sumatra’ mock-inoculated. The samples were harvested at 0, 1, 2, 3, 4, 5, 6 and 7 days mock-inoculated before RNA extraction.
- *Experimental sample* Pool prepared from root, stem and leaves from *N. tabacum* cv. ‘Sumatra’ inoculated with *R. solani*. The samples were harvested at 0, 1, 2, 3, 4, 5, 6 and 7 days post-inoculation before RNA extraction.

Five replicates for each time-point and library were prepared and used. Total RNA was extracted using the RNeasy kit (Qiagen, Maryland, USA) according to the manufacturer’s instructions. The Super-SAGE library was obtained using the protocol from Matsumura et al. [20]. The samples were sequenced with an Illumina Genome Analyzer II.

#### Identification and functional annotation

The libraries were normalized to 100,000 tags and the fold-change (FC) values for each tag was calculated by dividing the number of tags in the experimental sample library by the number of tags in the control library. Tags were considered differentially expressed if they exhibited an FC equal or > 2.5. The tag identification was developed using Blastn in the Sol Genomics Network (solgenomics.net) database [21, 22]. To indicate homology between tobacco sequences and database sequences, all *E* value scores less than 10^−5^ was considered significant and used. The Blast2GO software (http://www.blast2go.com) [21, 23] was then used to assign functional annotations to UniTags.

#### qPCR analyses

Total RNA was extracted from leaves, stems and roots from *N. tabacum* cv. ‘Sumatra’ plants inoculated and mock-inoculated with *R. solani* at 0, 1, 2, 3, 4, 5, 6 and 7 days post-inoculation using RNeasy kit (Qiagen, Valencia, CA). The cDNA were synthesized using the SuperScript III reverse transcriptase kit (Invitrogen, Carlsbad, CA). The 26-bp SuperSAGE tag sequences were used as 3′-RACE PCR primers with the primer polyT anc (Additional file 5).

Quantitative PCR was carried out using the QuantiTect SYBR Green PCR kit (Qiagen, Maryland, USA) with a Rotor-Gene 3000 PCR machine (Corbett, Australia). The qPCR conditions were an initial 95 °C denaturation step for 15 min followed by denaturation for 15 s at 95 °C, annealing for 30 s at 60 °C, and extension for 30 s at 72 °C for 40 cycles. The delta Ct method was used to calculate the relative expression, where delta Ct = (Ct of target gene − Ct of housekeeping gene) [24]. Results were based on the average of three replicates reactions per sample and three biological replicates. The significance of treatment effects was analyzed with as normally distributed data. One-way analysis of variance with post hoc pairwise least significance difference comparisons (P < 0.05) was evaluated using Statistical Package for the Social Sciences (SPSS 11.0, SPSS, Inc., Chicago).

#### Virus induced gene silencing (VIGS)

Virus-induced gene silencing (VIGS) was used to down-regulate the subset of differentially expressed UniTags. For VIGS analysis in *N. tabacum* cultivar “Sumatra”, a vector based on tobacco rattle virus (TRV) was used [21, 25]. A fragment of the phytoene desaturase gene (*PDS*) was used as a control to monitor silencing efficiency. Fragments of *NtPDS* and the group of differentially expressed UniTags were cloned using the *Sma*I sites of pTV00. The primers used are listed in Additional file 5. The TRV was infiltrated in *N. tabacum* using a total of 50 plants per construct and using the protocol developed by Ratcliff et al. [25]. After TRV infiltration, the accumulation of transcripts was evaluated using qPCR according to the procedure described above. Fifteen days post agro-infiltration, the plants were inoculated with *R. solani* and development of symptoms was analyzed 7 day post-inoculation. The evaluation was conducted in a randomized complete block design with the plants showing significantly lowered transcript levels [21]. Approximately, ten plants per replicate and three replications were used per experiment. An arcsine transformation was performed on all percent incidence data before statistical analysis to improve homogeneity of variance. Data were analyzed by analysis of variance or general linear model procedures of SAS (SAS Institute, Cary, NC, USA). Significant difference among means was determined by Fisher’s least significant difference mean separation at P < 0.05.

## Limitations

Basically, there are not resistant tobacco varieties into the commercial germplasm. Some species related show a significant resistance, however the procedure to obtain new varieties with a potential resistance might take long time. Regarding our study, the over-expression of down-regulate genes was not envisaged and this information might reveal new finding about the positive regulator genes of resistance.

## Additional files

**Additional file 1.** List of differentially expressed UniTags with GO categorization. List of differentially up and down regulated unitags.

**Additional file 2: Table S1.** Summary of all the analyzed SuperSAGE libraries.

**Additional file 3: Table S2.** List of the up and down regulated annotated tags with highest fold change of tobacco plants inoculated with *Rhizoctonia solani.*

**Additional file 4.** Relative expression of seven differentially up expressed UniTags in *N. tabacum* cv. ‘Sumatra’ plants at 15 days post-infiltration. Bars represent mean values and standard error of the results obtained from three replicates. A total of 30 plants with the lowest relative expression were used to calculate the disease incidence after disease susceptibility testing.

**Additional file 5.** Sequences of the DNA and oligos used from the up and down regulated annotated tags of tobacco plants inoculated with *Rhizoctonia solani* used during the real time PCR and VIGS analysis. Bold and underlined letters represent the “Tag”.

## Abbreviations

SAGE: serial analysis of gene expression
UniTag: unique Tag
AGs: anastomosis groups
CaM: calmodulin
JA: jasmonic acid
ET: ethylene
VIGS: virus-induced gene silencing
qPCR: quantitative PCR
