# Supplementary material for: High-throughput SuperSAGE for gene expression analysis of Nicotiana tabacum–Rhizoctonia solani interaction
Source: BMC Res Notes. 2017 Nov 21;10:603. doi: 10.1186/s13104-017-2934-9 (PMC5697063; doi:10.1186/s13104-017-2934-9)
Supplement: Supplementary file 2 — Additional file 2: Table S1. Summary of all the analyzed SuperSAGE libraries. [file 13104_2017_2934_MOESM2_ESM.docx]

**Table S1 Summary of all the analyzed SuperSAGE libraries**

| **Library** | **Mock-inoculated sample** | **Inoculated sample** | **Total** |
| --- | --- | --- | --- |
| Sequenced tags | 1,436,124 | 1,402,115 | 2,838,239 |
| Number of unique transcripts (UniTags) | 332,110 | 317,421 | 649.531 |
| Number of non- singletons | 104,517 | 124,113 | 228,630 |
