# Supplementary material for: High-throughput SuperSAGE for gene expression analysis of Nicotiana tabacum–Rhizoctonia solani interaction
Source: BMC Res Notes. 2017 Nov 21;10:603. doi: 10.1186/s13104-017-2934-9 (PMC5697063; doi:10.1186/s13104-017-2934-9)
Supplement: Supplementary file 3 — Additional file 3: Table S2. List of the up and down regulated annotated tags with highest fold change of tobacco plants inoculated with Rhizoctonia solani. [file 13104_2017_2934_MOESM3_ESM.docx]

**Table S2 List of the up and down regulated annotated tags with highest fold change of tobacco plants inoculated with *Rhizoctonia solani***

| **Tag code** | **Tag sequence** | **Protein description *** | **Accessions** | **Fold Change **** |
| --- | --- | --- | --- | --- |
| Tag_86182 | CTTATGGCTAGTTGTACCAAAG | Jasmonate ZIM-domain protein 3b (*Nicotiana tabacum*) | AGU37272.1 | 45 |
| Tag_50243 | AAGCAGAAATCAACGGGAGGAA | Receptor-like cytosolic serine/threonine-protein kinase RBK1 (*Nicotiana tomentosiformis*) | XP_009615440.1 | 38 |
| Tag_5425 | ACCAAAATTAGGCCTCAGAGAC | mRNA inducible by salicylic acid (*Nicotiana tabacum*) | AAA34120.1 | 28 |
| Tag_70806 | AATTGGCGCTGCATAGAAGCTG | DNA methyltransferase 1-associated protein 1 (*Nicotiana sylvestris*) | XP_009798987.1 | 17 |
| Tag_83440 | CTCCGAAAGAGCCTCACTGTTC | Auxin-repressed 12.5 kDa protein-like (*Nicotiana tomentosiformis*) | XP_009616586.1 | 16 |
| Tag_90374 | AGTTTAAAATATGAATTATTTA | BRI1 kinase inhibitor 1-like (*Nicotiana tomentosiformis*) | XP_009608327.1 | 13 |
| Tag_44413 | GATGTGGAGTGGAAGAGAATGA | DELLA protein GAI-like (*Nicotiana sylvestris*) | XP_009798071.1 | 12 |
| Tag_559 | AGTGCAAGCGTTCGAGGTTCCT | Xyloglucan endotransglucosylase / hydrolase protein 15 (*Nicotiana sylvestris*) | XP_009758414.1 | -53 |
| Tag_457 | CATGGCAATGGTGTTTGGCTGA | Pectinesterase / pectinesterase inhibitor U1 (*Nicotiana sylvestris*) | XP_009762395.1 | -44 |
| Tag_23336 | GTGCCCACTCTCTGGGAGGAGA | Auxin-responsive protein IAA13 isoform X4 (*Nicotiana tomentosiformis*) | XP_009621770.1 | -16 |

* Encode proteins were deduced by BLAST search. ** The libraries were normalized to 100,000 tags and the fold-change for each tag was calculated by dividing the number of tags in the inoculated sample library by the number of tags in the mock-inoculated sample library.
