# Supplementary material for: High-throughput SuperSAGE for gene expression analysis of Nicotiana tabacum–Rhizoctonia solani interaction
Source: BMC Res Notes. 2017 Nov 21;10:603. doi: 10.1186/s13104-017-2934-9 (PMC5697063; doi:10.1186/s13104-017-2934-9)
Supplement: Supplementary file 4 — Additional file 4. Relative expression of seven differentially up expressed UniTags in N. tabacum cv. ‘Sumatra’ plants at 15 days post-infiltration. Bars represent mean values and standard error of the results obtained from three replicates. A total of 30 plants with the lowest relative expression were used to calculate the disease incidence after disease susceptibility testing. [file 13104_2017_2934_MOESM4_ESM.docx]

**Relative expression of seven differentially up expressed UniTags in *N. tabacum* cv. ‘Sumatra’ plants at 15 days post-infiltration.**

Note: Bars represent mean values and standard error of the results obtained from three replicates. A total of 30 plants with the lowest relative expression were used to calculate the disease incidence after disease susceptibility testing.

Phytoene desaturase gene (*PDS*) (*Nicotiana tabacum*)


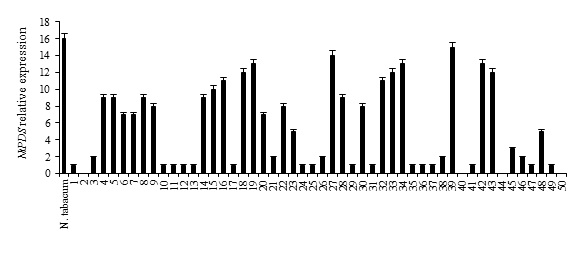


Jasmonate ZIM-domain protein 3b (*Nicotiana tabacum*) GenBank: AGU37272.1


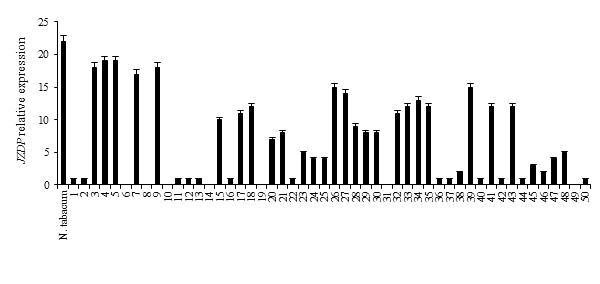


Receptor-like cytosolic serine/threonine-protein kinase RBK1 (*Nicotiana tomentosiformis*) NCBI Reference Sequence: XP_009615440.1


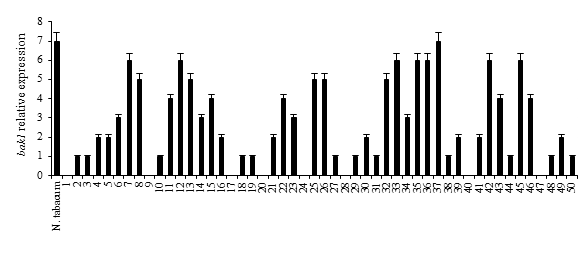


mRNA inducible by salicylic acid (*Nicotiana tabacum*) GenBank: AAA34120.1


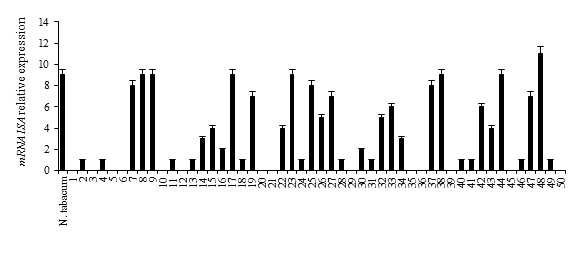


DNA methyltransferase 1-associated protein 1 (*Nicotiana sylvestris*) NCBI Reference Sequence: XP_009798987.1


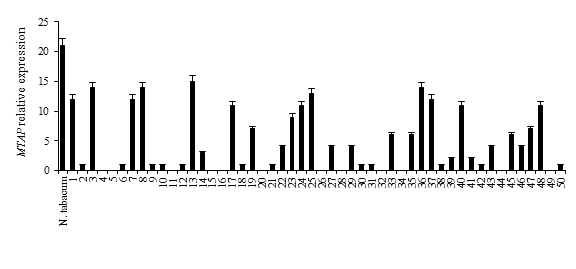


Auxin-repressed 12.5 kDa protein-like (*Nicotiana tomentosiformis*) NCBI Reference Sequence: XP_009616586.1


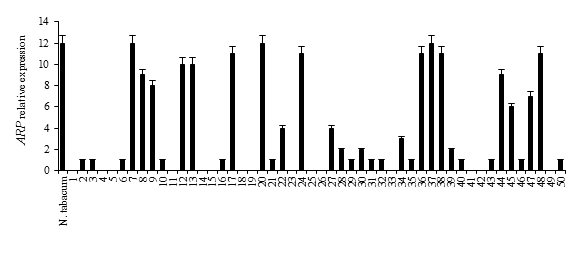


BRI1 kinase inhibitor 1-like (*Nicotiana tomentosiformis*) NCBI Reference Sequence: XP_009608327.1


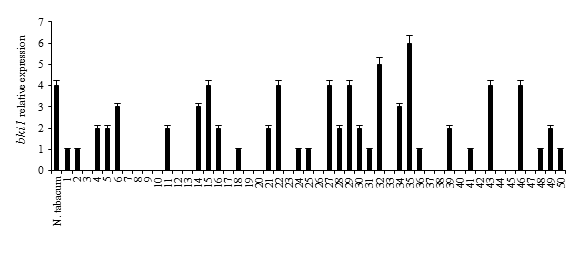


DELLA protein GAI-like (*Nicotiana sylvestris*) NCBI Reference Sequence: XP_009798071.1

**
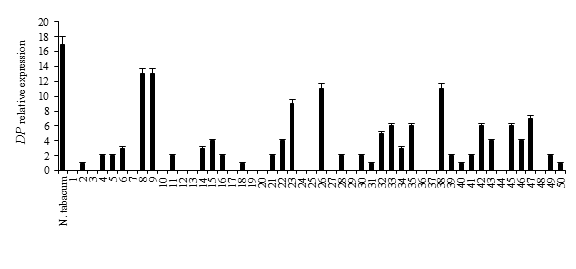
**
