# Supplementary material for: High-throughput SuperSAGE for gene expression analysis of Nicotiana tabacum–Rhizoctonia solani interaction
Source: BMC Res Notes. 2017 Nov 21;10:603. doi: 10.1186/s13104-017-2934-9 (PMC5697063; doi:10.1186/s13104-017-2934-9)
Supplement: Supplementary file 5 — Additional file 5. Sequences of the DNA and oligos used from the up and down regulated annotated tags of tobacco plants inoculated with Rhizoctonia solani used during the real time PCR and VIGS analysis. Bold and underlined letters represent the “Tag”. [file 13104_2017_2934_MOESM5_ESM.docx]

**Sequences of the DNA and oligos used from the up and down regulated annotated tags of tobacco plants inoculated with *Rhizoctonia solani* used during the real time PCR and VIGS analysis.**

Note: Bold and underlined letters represent the “Tag”.

*Nicotiana tabacum* phytoene desaturase mRNA

NCBI Reference Sequence: XM_016642616.1

TGGCTATGGTAGGAATAGCCAATCATTTTTGGGAGTCTAGCCAAACATAAAGGCCGGTCCAGTGCGAGTTACTGCAAATTGAGTTTGGAGTGAGGATTAAAGGAAGATAACATATTTCCAGCTAAATAGCAAACAAATGACCCATTAACGGAAGTGGCCAAACCACCAAATTCAGGCATCTCCACCAAATATTAGTTTTTTATACACAAAAGATTCAGCAATTCTTTATCAGGGGTATCTTTTTGTGGGTAACTGCCAAACCACCACAAATTTTCAGTTCCCACTCTTAACTCTTTTAACTTCAACACAACAACTTATTTGCTTTTCCTTCTTTGCTTATCTTGTGCATAACGATTTCCTACAACTTTAGCATAATCTTGGTTTGTAATCCACAACGTGAAACACAACTCCTAGGCGGTTTCATACCGAGGCTTAATTTACTGCTATTTTGTTCAGTAAAATGCCCCAAATTGGACTTGTTTCTGCCGTTAATTTGAGAGTCCAAGGTAATTCAGCTTATCTTTGGAGCTCGAGGTCTTCTTTGGGAACTGAAAGTCAAGATGGTCACTTGCAAAGGAATTTGTTATGTTTTGGTAGTAGCGACTCCATGGGGCATAAGTTAAGGATTCGTACTCCCAGTGCCATGACCAGAAGATTGACAAAGGACTTTAATCCTTTAAAGGTAGTCTGCATTGATTATCCAAGACCAGAGCTAGACAATACAGTTAACTATTTGGAGGCGGCGTTATTATCATCATCATTTCGTACTTCCTCACGCCCAACTAAACCATTGGAGATTGTTATTGCTGGTGCAGGTTTGGGTGGTTTGTCTACAGCAAAATATCTGGCTGATGCTGGTCACAAACCGATATTGCTGGAGGCAAGAGATGTCCTAGGTGGAAAGGTAGCTGCATGGAAAGATGATGATGGAGATTGGTATGAGACTGGGTTGCACATATTCTTTGGGGCTTACCCAAATATGCAGAACTTGTTTGGAGAACTAGGGATAAACGATCGGTTGCAGTGGAAGGAACATTCAATGATATTTGCGATGCCTAACAAGCCAGGGGAGTTCAGCCGCTTTGATTTTCCTGAAGCTCTTCCTGCGCCATTAAATGGAATTTTGGCCATACTAAAGAACAACGAAATGCTTACGTGGCCCGAAAAAGTCAAATTTGCTATTGGACTCTTGCCAGCAATGCTTGGAGGGCAATCTTATGTTGAAGCTCAAGACGGTTTAAGTGTTAAGGACTGGATGAGAAAGCAAGGTGTGCCTGATAGGGTGACAGATGAGGTGTTCATTGCCATGTCAAAGGCACTTAACTTCATAAACCCTGACGAGCTTTCGATGCAGTGCATTTTGATTGCTTTGAACAGATTTCTTCAGGAGAAACATGGTTCAAAAATGGCCTTTTTAGATGGTAACCCTCCTGAGAGACTTTGCATGCCGATTGTTGAACATATTGAGTCAAAAGGTGGCCAAGTCAGACTAAACTCACGAATAAAAAAGATTGAGCTGAATGAGGATGGAAGTGTCAAATGTTTTATACTGAATAATGGCAGTACAATTAAAGGAGATGCTTTTGTGTTTGCCACTCCAGTGGATATCTTCAAGCTTCTTTTGCCTGAAGAGTGGAAAGAGATCCCATATTTCCAAAAGTTGGAGAAGCTAGTGGGAGTTCCTGTGATAAATGTCCATATATGGTTTGACAGAAAACTGAAGAACACATCTGATAATCTGCTCTTCAGCAGAAGCCCATTGCTCAGTGTGTATGCTGACATGTCTGTTACATGTAAGGAATATTACAACCCCAATCAGTCTATGTTGGAATTGGTATTTGCACCTGCAGAAGAGTGGATAAATCGTAGTGACTCAGAAATTATTGATGCTACAATGAAGGAACTAGCAAAGCTTTTCCCTGACGAAATTTCGGCAGATCAGAGCAAAGCAAAAATATTGAAGTATCACATTGTCAAAACTCCAAGGTCTGTTTATAAAACTGTGCCAGGTTGTGAACCCTGTCGGCCCTTGCAAAGATCTCCTATTGAGGGGTTTTATTTAGCTGGTGACTACACAAAACAGAAATACTTGGCTTCAATGGAAGGTGCTGTCTTATCAGGAAAGCTTTGTGCCCAAGCTATTGTACAGGATTACGAGTTACTTCTTGGCCGGAGCCAGAAGAAGTTGGCAGAAGCAAGCGTAGTTTAGCATGGTGAACTAAAATGTTGCTTCTGTACACTAAATTTAAGATGAAGGCGGCCACACTGAATTAGCGTTGTACACAACATATACAAGGACAGTACAACATTGAACCCAAATACGAGAAATGTTACACAAATATGAAATATGTGCTCTGCTTTCCCTCCGA

Primers used for construction of VIGS vectors:

TTCTTCAGGAGAAACATGGTTCAA

TCCACAATCGGCATGCAA

Primers used to evaluate gene silencing efficiency VIGS-silenced plants:

CTTTGGAGATCTCAGCCTTGGT

CGCCGTAGAATATCGTCATCTG

**Tag code: Tag_86182**

*Nicotiana tabacum* jasmonate ZIM-domain protein 3b mRNA, complete cds

Encode proteins were deduced by BLAST search: Jasmonate ZIM-domain protein 3b (*Nicotiana tabacum*) GenBank: AGU37272.1

ATGGTGGATTCCGGTAGATTCGCCGCCGCCGTCGGTCAGAAATCACATTTCTCTCAGACATGTAATTTGTTGAGCCAATACTTGAAAGAGAAGAAAGGTTCCTTTGGAGATCTCAGCCTTGGTATTCACCGCGCTGGCACTACTACTATGGATTTGTTGCCAATGATTGAGAAATCTGGTGAGTCAAACCCTCAGAAATCAATGTATCTGTTTCCTCAAACTGAAGCAAAATCTGAACCGGAGAAAGCGCAGATGACGATATTCTACGGCGGTCAAGTTATTGTGTTTAATGATTTTCCGGCAGATAAAGCTAAGGAAATCATG**CTTATGGCTAGTTGTACCAAAG**GAAACAACAACAGTACTACTCAGATTCAAAAAACAGCTGAATCTGCTTCAGATTTGGTGCCTCAGCCTATTATTTCTGGAGATTTACCAATTGCGAGACGAGCTTCACTTACTCGGTTTTTGGAGAAAAGAAAAGATAGGCTGACTGCAAAAGCACCTTACCAATTAAGCAACCCAAATAAACAGGTAGCAGTTTCTGAAAACAAGGCGTGGTTGGAATTGGGTGCTCAATTTCCAGTGAAAACTGAGCAATTCTAG

Primers used for construction of VIGS vectors:

TTGGAGATCTCAGCCTTGGT

CCGCCGTAGAATATCGTCAT

Primers used to evaluate gene silencing efficiency VIGS-silenced plants:

CTTTGGAGATCTCAGCCTTGGT

CGCCGTAGAATATCGTCATCTG

**Tag code: Tag_50243**

PREDICTED: *Nicotiana tomentosiformis* receptor-like cytosolic serine/threonine-protein kinase RBK1 (LOC104108161), mRNA

Encode proteins were deduced by BLAST search: Receptor-like cytosolic serine/threonine-protein kinase RBK1 (*Nicotiana tomentosiformis*) NCBI Reference Sequence: XP_009615440.1

TCTTCTTCTACCTCTCTTTTCTTCAAATAAGAAAAACAACTTCTCTAAGATATACCATTTTCTCTTCTTCTTCTTCTTCTTCTTCTTAGGGGAGAAGAATTATGAATTATGATTGAACCTCAAATGTAAACCTTCATAGAATATGTTCTAATTCTTCTAAATTCCTTTTCATTTGCTTCATAGCAAATTCTTCAAATTTATTCATTTGTCTTTAACACAACATCTACAAATATACCATGGAAGGTGAAAGTGTTGTACAAGTTCAAGAAAATGGAGCAAAAGAAGATATGAAGAGAGAAGAAGCAGAAAAGGAACAAGAAGAAGTAGAAGTAAAAGTAGAAATAGAAATTTTGGAGGGTAAAGAAGAAAATGGAGAAAAGGACAAAAAAACCAAAGATGATCAGTCATCACCAAGGGCAGTTTTGGAAATTCATATCTCAGGTACATCAGATTCCGACAACAGCAGCATTAGCAGCGGGGAAAGGAGCTGCAGTTTCGGGTCATCTCCGTCTCCCGGCGGCGAAAAATCGGCAGTTTCCGGCGGCGGAGGTGGCGGAGGTGGAGGAGGAGAGGAAGCGGAGCAAGGATTGAGTTTTAAGAATTTTTTTGATCAAATGAAGAAAAAGTCTATAAGAAGATTATCGACTATACAATTATTTGGAGGATATGAAAATTTACTACCAAAGAAAAATATTAAGAGGAAATTACTGGCTAGAATTCGAAGTGCAGAAGAAGATAGAATTGATTGCCATGATTTTGTTGTGCCTAAGCCTTCTTGGAGGAATTTTAGCTTTGATGAACTTGCTCAAGCTACTGATAATTTCACTCCAGATAACTTAATTGGCAAAGGAGGACATGCAGAAGTGTACAAAGGACATTTACCTGATGGACAAGTTGTAGCAGTCAAGAAAATAACAAAGAAAGAAAAGAATGACGAGGACAGAGTTGGAGACTTCTTATCTGAGCTAGGAATCATTGCTCATATCAATAATCCTAATGCTGTTAAGTTAATTGGTTTCAGTGTTGATGGTGGTCTGCACCTTGTTCTTCAATACCTGCAACATGGCAGCCTTGCCTCTGTACTGCACGGTAGAGAAGAGTGCCTTGAATGGAAAATAAGATATAAAGTGGCAGTTGGAGTAGCTGAAGGATTGCGTTATCTTCATTCTGATTGCCAAAGGCGCATAATCCATAGAGATATTACAGCATCAAACATTCTACTTACTGAAGATTATGAACCTCAGATATCTGATTTTGGACTTGCAAAGTGGTTGCCAGAAAAATGGGCTCATCATATTGTTTCCCCAATTGAAGGAACTTTTGGATATATGGCACCAGAGTACTTTATGCATGGAATTGTTCATGAGAAGACCGATGTTTTTGCCTTTGGAGTTCTGCTATTGGAGCTTATTACTGGTCGTCGTGCTGTTGATTCATCCCGACAGAGTCTTGTGATGTGGGCAAAACCGATGCTGGAAAAGAACAACATCAAGGAATTAGCAGATCCTCGTCTGCTCGATACCTATGATGTTATTGAGATGAAACGAGCCATGTTTACAGCGTCTACATGCATTCACCACTTGCCAAACATGCGTCCCAACATGAAACGGGCTGTTCAGCTGTTGAAAGGCGAGAACGAACCCATAGACATG**AAGCAGAAATCAACGGGAGGAA**GATCATTGATGCTGGATGCTTGTGATTTAGACGATTACAGTAACACAACTTATCTCAAAGATCTCAATCGTCATATGCAGCTCGTTATGGAGTAATCTTTTGATCTTATGATCAAGTCGCATTGATCCTTGTTCTGCATCTGTTGCTGTTAGCTATTCGTACTTGTTCTCTGTTTGTACATAAATCTTGTAAAATCAAGAGCTCAATCAAGTCACATATTCCACTAGCTGTTTTTTGGAGTGAAAGGTTTTGATATGCTGTCCATCTTAACACTGAGAAGCAGCATCGCAAACATCTAAGTTTATCTTCTAATGTGTCAACTGTTGATTTGCCAAGAACGAAAAAAGACATTAGCAGCACAAAATTGATGCAGCAAAAAGCAAGTGATTATACTGCATTGTCACTTACTCATCAAAATGATTGAAAGAGCTTATTTCA

Primers used for construction of VIGS vectors:

AGTGGTTGCCAGAAAAATGG

TCTGTCGGGATGAATCAACA

Primers used to evaluate gene silencing efficiency VIGS-silenced plants:

AGAGTCTTGTGATGTGGGCAAA

CTGCTTCATGTCTATGGGTTCG

**Tag code: Tag_5425**

*Nicotiana tabacum* Sar8.2k gene, complete cds

Encode proteins were deduced by BLAST search: mRNA inducible by salicylic acid (*Nicotiana tabacum*) GenBank: AAA34120.1

CATGTGAGAAACCACTCACCCAACTAACTCAATCAACCCTGGCGGCGGCGTGACTTCACAGTAGATCTTTGTGAAGGCTTTCTTTTTGTCATTTTTCTCCTTTGTTTAGAACAAGTTGTTCTGACAGTTGGACGAGAAACTATTAAGTTCTATAAATAGGGGAGAAACATTGTTTTCCTTTTTACAGCAAAATATTGAAACTCCAAATAGCTCATCAAAATGTTTTCCAAAACTAACCTTTTTCTTTGCCTTTCTTTGGCTATTTTGCTAATTGTAATATCCTCACAAGCTGATGCAAGGGAGATTTCTAAGGCGGCTGCTCCAATTACCCATGGTTTATTTACTTCTTCAATCTATTACTACTCCAGTAGTTATTTTCATTTTTGACATTTTGAGTCTGCAAACGAATGCTATAAATGTTTAAATATTCATGTAACATATTTCTTTGAATTTTATGATCTTAAATATGTCATAATATCTTATACAGAAGTCTTATTAATGAAAAAAATCGGAATTTCGGAATTAAAAAAGTTATTAAATGTTGGTGGCATTCTTTGTTGAGATGACTTAAAAGGAAAGCAAAACACATGATTGAAACGAAGGGAGTAAATGTTTACATAAGTATTATTTAACATTTTGCTGAAAGTTTAATGTCGTTTTGAAACTTTCTGAGACATTCTACATCTATAATATTTAATAATCTAAGGATATCGTTCCAACACCGCAGAAGTTTCTTCTTATCTTTATTGAATTTGGTGATTATTACCTCGTTTAAATTCTATTGCTAGAGTAAGGACATTCAATTTAATTATTTTTATGTCTGCAACATGCATTCTTACATGCGGCCATGATTCTTTCTTCTTGGCCAAACACGTGAAAATATCTTTCTTTTGGATGGCGTTAAGATTTGAATATTCGAAACCTTGTTTTGGCCAATTGTGTGATCAATAACAATAACAACAAACTTAGAATAATTTCACAAGTGGGGTATCGAAAGAATAATGTTTATGCAAATCTTACCCTTGTCTTGAGAACTAGATAGACTGTTTCCGATAAACCCTCCGACTCCAAGAAAAGGATGAAAAGGCAGTTAGAAATTAAATAAGCCAGTAATACCAGCAAGATAATAAAATAATCCGAACCAAAAGAATACCGTGGGATCCGCTGATGCAAGGGAGATGTCTAATTCTCGACCTTCACGCCCTCCTATCAAGGGTCATGTCCTTACTATTTAGGATAGAAGGTTAGTGGGACAAGACAGATGCACGACTACCTACTAACTTTCTATCCTAATTCTCGAACTCCACGTCCTTCTATCAAGGGTCATTCCCCGGTAAGCTGAAGCAACGCCATATCCTGTCAATTGTGTGATCATTTTAATTAAAAATTAAGCTATTAGAGGAGGAACCTAAATTCATCCTAATTTATTTATGTATGCAGCACTGAGAATTAATATGGTTACAAATTAATTTACTCCTTTATTGGTTAACCTATAACTAATACATTACTCTACATAACCTAATTTGATGCAGCAATGGATTCAAACAACATTACTAATCAGAAGACGGGTGCCGGAATCATCCGTAAGATACCGGGTTGGATACGAAAAGGTGCAAAACCAGGAGGCAAAGTCGCCGGCAAAGCTTGTAAAATTTGCTCATGTAAATACCAGATTTGCAGCAAATGTCCTAAATGTCATG**ACCAAAATTAGGCCTCAGAGAC**TATGTACTTGTGCTGGTGTGAGTTTAGTTTTGAGAATAAAGGGAAAGTTATGAATAGCCTAATATAATTCTATTCACTTTCCTCTAGTTAATTTCTCTTAGTTTGTGTTTTGTTTTGTTAGTAGTTCCTATTGTTGCAACTTGCAACAAGTCTTGGGTCAACATGTACCTCTTGTCTTGTAGTCTTTCGACTGTATGATATTGTACCGTATTGTATTGTATTGTATTTTTTCTTTAGCCACTTGTTATCTGAAATCAAATCCAATTAAATCTAATTCGCGTTGC

Primers used for construction of VIGS vectors:

TCCGAACCAAAAGAATACCG

GACAGGATATGGCGTTGCTT

Primers used to evaluate gene silencing efficiency VIGS-silenced plants:

TGGCCAAACACGTGAAAATATC

TTTCTTGGAGTCGGAGGGTTTA

**Tag code: Tag_70806**

PREDICTED: *Nicotiana sylvestris* DNA methyltransferase 1-associated protein 1 (LOC104245118), transcript variant X2, mRNA

Encode proteins were deduced by BLAST search: DNA methyltransferase 1-associated protein 1 (*Nicotiana sylvestris*) NCBI Reference Sequence: XP_009798987.1

TTCGCGCTCAAAAAGGGGCAAAGGATCATCGCTCTTACTTGTTCCTTGAATGATAATCCCATTCGTGTGTAGACTAATTCAGCTGTTCTATTTTCCAATTCTTCAACGTTTGTACTAATTTATCATATCGTACTTCAATATTAGTACTACAGAGTGTTAGAGCAAACCCTAATTTCGTAGAAAATAAATCCATATGGATGCGAAGGACATCTTAGGATTGCCCAAAAATGGACCCATTATGTCCCAAGAAAAGAAATCGAGGCCTCAGAAAGAATCTCAAAGGAAACCTGATGGCATTTCGCGAGAAGTTTACGCACTTACAGGTGGTATTGCACCTCTCATGCCTTCTCTTGATATTAATCAATTGAAACGAAAAGCTCTTTCAGAGTCCGAAAAGATTACATGGCAATGGCTTCCTTTCACAAGCTCTGCTAGAAAAGATAACTTACAGCTTTATCATTGGGTGAGAGTTGTTAATGGTGTTCCGCCTACAGGTGACTATTCGTTTGCCAAGTTTAATAAGTCTGTGGATGTGCTTGAGTACACTAACGATGAGTATGAGAAGTTCTTGTCCGATCCTTCATGGACCAAGGAAGAGACTGATCAATTATTTGAGCTGTGCAAGAGGTTTGATCTCCGTTTCATTATCATAGCTGATAGGTTCTCTTCAAATCGTACAGTTGAGGAGCTAAAGGACCGCTACTACAGTGTATCTCGTGCTATTACAACTGCTAGGGCTGCATCCCCTGCCGATGTTGCTGGACATCCCCTTGTAAAGGAGCCTTACAATGTCTCTCAAGAGAGAGAGCGAAAACGTGCACTGTCGATGGTGCTTTCACAGACAAAGCTGCAAGAGCGGAGGGATGCAGAGGTTCTTGCCGAAGCAAAACGAATATCAGTTGCACGAAAATCTGTGAAGGCAGCTGAAGAGACAGAACTTCCAGTTGTGTCCGATGCTGGTCCTGAAGGTGCTGAAAAAGCTGCCGGTATTGATGGAATATCAACCTCCCCAAATGCTCAATTACCCTCTGGGTTTGCTGCACCTCCAGTGTCAGAGACTGCTTCTACTTTGGCTTCTCTTCGCATGCTGCGTGTGTACTTGAGAACATATGCACTAGACCAAATGGTGCAAGCCGCGAGCTCTTCAGCTGGACTTCGGACAATCAAGAGGGTCGAACAAACTTTACAAGATCTTGGGGTTAATTTGAAGCCAAAGGTCCCAACTAAGCTTGTCTGTGCGGAGCATCTTGAACTGAGGAAAGAAATACTAACCCTGTTAAATCTTCAAAAGCAGCTACAATACAAGGAGGCAGAAGGCTCATCTTATCGTGAAGGTTCATATTCTGAGACTCCTGGTACACCTCCTAAGCGTGCACAACAAGACAGAACATTCATCCCCGACTCTACAAGTTTTGGAGGGGACAGAGTTGGTAAAAGGGACCAGAAACGCAAGGGGCCTGGGCGATTATCAGAAGTTCCATCTTCGCCAGCACAGTCAAAACGGCCCCGCAAGTTAAAGACTTCAGATGGATGAATGGGAGGGTTAGTACATTTTTCCTCTCAATGCTCTTAATGTTGTGTACGTCAGAAAGCTACAAGGAGCTGTGGTAAGCTGCTTGAATCAACTTCTGGTGCCACTGTGATGTGTTCTATGTCTATTTGGATTTCCTCCAGCATGAAACGTGATCGAACCTTGCAAATCTCAAATGTGGCTCTCTTTTAAGTGACACAAGAAACGTGAGATGCCCCTATAATGCTTTCTGTGGAGATCAGTCCTCCTTTGCTTCAGAGTACAGTCCACGCAGTCTGTAAATACATCTGTAACTTAAAATTGTGATAAGCATGTTAGCCTCCGCTTTCTACCATATCTGTTTGACATGTCATTAAATTATTGAGCCATGTTGGCTTTTTTTTTTTTTGTATTAACACATCGAAGTAGTTGTGGTTTAGTTGCGTTTAAGTTGCTTACTGGAATGGACAGACATG**AATTGGCGCTGCATAGAAGCTG**AAAGTGAAAAGGGCAAGCGATCTGCTTCTTTGGGCCCTCATCACGAATCATAATAGTTATGGTGCTGCTGCTTCTCTTTTTTGGATTATCTTCTTTTCCTATTTGTATATGGTTCTTGTATTTGATGGATGTAAAAGTAGATAGTACTGTATTTACATGTGTCCCAGACCTGATTTAGTTTCTACACTCAAATTTGCAGTA

Primers used for construction of VIGS vectors:

GGTGCCACTGTGATGTGTTC

TGGTAGAAAGCGGAGGCTAA

Primers used to evaluate gene silencing efficiency VIGS-silenced plants:

GAAATCGAGGCCTCAGAAAGAA

AGGCGGAACACCATTAACAACT

**Tag code: Tag_83440**

PREDICTED: *Nicotiana tomentosiformis* auxin-repressed 12.5 kDa protein-like (LOC104109088), mRNA

Encode proteins were deduced by BLAST search: Auxin-repressed 12.5 kDa protein-like (*Nicotiana tomentosiformis*) NCBI Reference Sequence: XP_009616586.1

CCATTTAGTATTTACACTACAGACTAAGAAAAACTTAAGACTGACTACAGAAGATCATAAGGAGAGAAGATGGTGTTGATTGATAAACTATGGGATGATGTTATGGCTGGACCTCAACCTGATAAAGGCCTTGGCATG**CTCCGAAAGAGCCTCACTGTTC**AAACTGGTGGGGAATCTGGAGAAGGATCTAGCAAGTACCAGAGGTCTCTCTCGATGCCGGCCAGTCCGCCGACGCCGGCGACACCAACAACTCCGTCACCGACGGCAAGTAAAGAAAACGTATGGAGGAGTGTTTTTCACCCAGGAAGCAATATTGCCACAAAGAGAATTGGTGCTCAAGTCTTTGACAAGCCCTCTCACCCTAACGCTCCCACTGTTTATGACTGGCTCTACAATGGGAACACCAGATCTAAGCATCACGAGAAGTGCTGAGAGTTTTTGGGTGGCAACAAGTTGAAACCTATGTAAATAGTGTTTTTTCCGGCGAGTTGCTTCACTAATGAAGCATGGATCACTGGACTTTTTATCTTTATTTCATATTATATGAATTTCTGTTGGGTATTAAGCAACAAGTATGTTATAGTTAGGTTTTGCAAGCTAACTTGAGCTTTAATGGCCATCAGTTGCTGCTGCTACTACTGTATGTTTGTGGTAGACCGCTATTTTGATGTTTGTTTGTGTATAATTAAATCTCTATTATAAATATTGTGATGTTATTGTTTTGTTATTA

Primers used for construction of VIGS vectors:

GGAGTGTTTTTCACCCAGGA

CTTGTTGCCACCCAAAAACT

Primers used to evaluate gene silencing efficiency VIGS-silenced plants:

AGGAGTGTTTTTCACCCAGGAA

AACTTGTTGCCACCCAAAAACT

**Tag code: Tag_90374**

PREDICTED: *Nicotiana tomentosiformis* BRI1 kinase inhibitor 1-like (LOC104102343), mRNA

Encode proteins were deduced by BLAST search: BRI1 kinase inhibitor 1-like (*Nicotiana tomentosiformis*) NCBI Reference Sequence: XP_009608327.1

ATCTCATAGCCACCACATAACCCCTTCTCTTCATTCAACAATAAACAATATTCATATCACTATAATTGCCACACAAAACTCTTTTGTATAAGACATTTTAGCCTAAATTCCCAACACCCCCTTCTTCACGTATCTTATCTTCTTCTTTTTTCTTTTTCTCCTCAATCAAAGAAAAACAAGAAAACTTGAAAAAAAGATCCAATATTAGATCTTAGTACTAAATTTATTACCCTTTTAAAACATTTTTAGTCATGGACAAGATGAGAGTCAAAGAAGAATTGAAGCAGCCCAATCAAGAAAGCCAAAAGGCAAAGCAAATACAACAACAACAACCACCGTCCACCACCGCCGCCGGCAGTGCAGCTGCAGCTTCACCACCTTCAGCTTCATCATCTCCATCACATGAATTTTCCTTCACAATTTCCTTTAATGAAAATAACTCAACAAAAACCCCTCTTGATAATAATAAAACAAAGCAAAATATTCCACCATCTTCTTTTGCTATAGATTTAACTCCAGCTGATGATATTTTTTTCCACGGCCATTTACTTCCTCTTCACCTTTTATCCCATCTTCCAGTTTCTCCTCGCTCTTCTACAAATTCCATTGATAGTTCACTTCCCATAAAAGACTCATTAGAAGAAAAGAAAATCCAAAATTCCAAAAACCAAAAAGAATTAGAACATGATGATGATAACTCTTATTATGATCTAAACCATAGCTTTCATCATCCTCATCAAACAAATTCTTTTACTACATCAAAAGATCAAAAACCAAAGTCCAAATCTTTTTCTTTATTTGGATTACCAAAAAGGAAAAAGGAGGAAAAAGAAGACAAGGAAAAACAAAGGAAGCTAAAATTTGATGTGAGTCAAGTCTTGAAAAGGTATATGAGAATGGTGAGACCTTTTTTATCTTTTAGAAGCAGAAAAAATATGCAATTACATAGACAAAGTTATTCTTATTCTGGGAATTTAAGTTTTAGAAGTGGGAAAAATAAGGAAATTAGAGGAATAAAAAGAGGTGCATATTCAGCTCCAGTATCTATGAAAACTTCTCCTACAAATAGTGGTCTACTTGTAGCAACACCAGGTGCTAATTATCATAATTCATCTTCAAGTGATAGTACTATGGAAGAATTACAAGCTGCTATTCAAGCTGCTATTGCTCATTGCAAGAAATCTAGTTCAGTTGAACAGAAAATCAAATGCCAAGAAAATGATTAGGTTATTGAAGTTCACTGGCTCGACTAATACAGATTCGCGTCGCTAAGGATAAAATGCTCCAGAAGTTACCTCTGGTTATAATTCGATCCTACCCCTTGTGGTATTTTTATCCTTTTTATTATTGCTTCTTTCTTGCATACTTAAAAATTGTTTTTTCATGTATCATAATCATGAAGTATATTTGTAAATAAAGTATTGTATTACATG**AGTTTAAAATATGAATTATTTA**TTTATGGTGAA

Primers used for construction of VIGS vectors:

CACCACCTTCAGCTTCATCA

GAAGTAAATGGCCGTGGAAA

Primers used to evaluate gene silencing efficiency VIGS-silenced plants:

CACCACCTTCAGCTTCATCATC

GAAGTAAATGGCCGTGGAAAAA

**Tag code: Tag_44413**

PREDICTED: *Nicotiana sylvestris* DELLA protein GAI-like (LOC104244356), mRNA

Encode proteins were deduced by BLAST search: DELLA protein GAI-like (*Nicotiana sylvestris*) NCBI Reference Sequence: XP_009798071.1

TTTAAGCGACTTAGGAGGGTGACTCCAACAATTTATTAAAAAAACAAAAAGAAGATGAGTTATAGATCAGAGATGTCTATGGACACAACCACGGAAGATGGGGAATCCTTTATTTCTAATACAGATGCAATCATCAACGGCACTTCTGATATTTCAGCGTTGGCTGAGAGCTTAATCTCAGGTCCCAACATCCCTAATTCGTCTTGGACGAGTGCAGACGACGTCCAGCAGCAGATTTCGACGGCTGGTGGTGGTGGTGCTGCTGCTGCTGATGATATGATGATTGTATCATCAGGTGCATCTTCCATGTTATCCAATACTAATAAGCAAAGTGAGATTATGATATTTAATGTTGATTTCAGGGCGGTTGCTGTTTGCAATAACAGTGATAAAATTGAGGAGAAAGGATCAGCTGAGAGCAGTAAGCGATTGGAATGTCGCTTTGGTTTAGGTTCGAATATTGGTGGGGCAGTGAATGAGGAAACCAGCTTTAGGTTTGTCAATATATATACATTGATGGCTTGTGCCGAGGCAATCCAACAGAACAACTTAAATCTAGCTGATGCACTCGTCAGCGACATAAAAAGACTTGCGGTTCAACAATCTGGAGCCGTGAAAAAGGTGGTGTATTATTTTGACGACACTTTGGATCGAACTATTAACGGAATGAATGCACCGTATATTGTTGAATCCTCCTATTGCACGGATGAACTCTTGCAGATGTACTTCTATGAGACTAGCCCCTACCTCAAATTCGCCCACTTCACTGCCAATCAAGCCATTCTTGAGGCCTTTGCCGATTCAAATAGAGTACATGTAATTGATTTCAGTTTGAATCAAGGTTCGCAATGGCCGGTGCTAATGCAGGCTCTCGCCTTGCGTCATGGGGCTCCACCGGCTTTTAGGCTTACCGGAATTGGACTACCACAACACTCCTTTTTGGAAGTCCGGTATATGCTAGTGCAGTTGGCTGAGAAAATAGGTTTAGAATTTGAATTCCGTGGATTCCTAGCCAATTCTTTAACCGATGTTGACGCTATGATTCTGAGTATTAGATCAAGTAATGTGGAAGCAGTGGCTGTGAACTCTATTTTCGAGCCTCACCATGCGATGTCCAGGCCAGGCGCAATTGAGAAATTGCTGAATACAATCAAAGAGATGCAACCGAAGATTGTGACGACCATCATCAAACAAGAAGCAACAAACTCAGAGTGGACTCAGTCCTACAGCCGAGACTCGGAGATGGCGGAGCAGTACCCAGGGCGGCAGATTCATAATGCGGTGACTTGCAAAGGGAGTGATCGAATTGAGAGGCACGACACTCTTAGTCAATGGAGAGTGAAAATGAACTCAGTTGGATTCAACTCGGTACGCTTGAATTCGAACACGTGCAACCAAGCAAGCAAGCTTTTGTCCTTGTTTCATAATAGGCATG**GATGTGGAGTGGAAGAGAATGA**TGGGTTTCTTATGTTGAGTCGGGATAGTCAACCGCTCATTGCCACCTCGGCTTGGCAGCTTACGCCTCCGACTCTAGGCTAGTGGGTCAACGGTTGAACTTATGAAATGTTTGGACAGTTTTTAGAGAAAAAACTTTCCTTTCTTCCAGAGGCAGTTTCTATTTCTGTTTACCTCGAAAAATATGAGTAACAATTAAAGATAATTTGTGATTTTAAAGA

Primers used for construction of VIGS vectors:

GATTGGAATGTCGCTTTGGT

TTTATGTCGCTGACGAGTGC

Primers used to evaluate gene silencing efficiency VIGS-silenced plants:

GGTGCATCTTCCATGTTATCCA

CCCCACCAATATTCGAACCTAA

**Tag code: Tag_559**

PREDICTED: *Nicotiana sylvestris* probable xyloglucan endotransglucosylase/hydrolase protein 15 (LOC104211083), mRNA

Encode proteins were deduced by BLAST search: Xyloglucan endotransglucosylase/hydrolase protein 15 (*Nicotiana sylvestris*) NCBI Reference Sequence: XP_009758414.1

TTCATATCATCAATCACCACACATTTGATTTCAGACATACCCCTTCTTCTTCCAACTTGAAAAAAACGTTGAGAAATCATTTTGAAAAATGTCGCCTCGTTTCTCTTTCAAAATGTTAATCCTTCCTATAGTCATGGCAAGTCTATGGGCAGCCGCCTCAGCTGGTAATTTTTATAATCTTGCAGATATCACTTGGGGCGAAGGACGTGGTAAAATAACAGAAGGAGGCAGAGGCCTCTCTCTGTCCCTTGACAAATTATCTGGTTCAGGTTTTCAATCCAAGAATGAGTATTTATTCGGAAGATTTGACATGCAACTCAAACTTGTCCCTGGAAATTCTGCTGGCACTGTCACCACCTTCTTTTTATCTTCACAAGGAGCAGGACATGATGAGATTGACTTCGAGTTCTTAGGCAATGTTTCTGGTCAACCTTACACAGTCCACACCAATGTTTACTCGCAAGGCAAAGGCAACAAAGAACAACAATTCCATTTGTGGTTCGACCCAACTGCTGCATTTCACACTTACTCCATCATCTGGAACGCTCAGAAAATCATTTTCTTGGTGGATAATAGTCCAATCAGAGTATACAACAACCACGAAAGCAATGGCATTCCATTCCCAAAAATCCAACCAATGAAAGTGTACTGCAGCTTATGGAATGCAGATGAGTGGGCAACACAAGGAGGTAGAGTCAAGACAGATTGGACACATGTTCCTTTCACTGCTTACTACAGAAACTTCAATATTGATGGCTGCGCAGTTACATCCGGCACCTCTTCGTGTAAGTCCACTGATTCAGCCAACAATGCTAGGCCATGGCAAAATCAAGAACTTGATGCTAAGGGCAGGAATAGGCTACGATGGGTTCAAAGCAGACACATGGTTTACAACTATTGTGCTGATTCTAAGAGGTTTCCTCAAGGCTTTTCTCATG**AGTGCAAGCGTTCGAGGTTCCT**GTAATTAAGTTGCTGGAACAAGGAAATTGTGGCACTGCCTCGGCTATTTATTAAGATTGATTCTTCTCCTATTTGTGAATAATGAATATATGTACTGAATTCTTTATTTATAAGATCAACATCCTTTATTTTTTATGTAACTGCTAATGTATTTGTTTTGGAGCATACACTGCACATGGATCGGAATAAAATCTCTTTAGTTTC

**Tag code: Tag_457**

PREDICTED: *Nicotiana sylvestris* pectinesterase/pectinesterase inhibitor U1 (LOC104214436), mRNA

Encode proteins were deduced by BLAST search: Pectinesterase/pectinesterase inhibitor U1 (*Nicotiana sylvestris*) NCBI Reference Sequence: XP_009762395.1

CTTCTCTTCACAACTCTACCTTATATTTCTTCATCCAAAATGACACGTGTCAAAGATTTCTTCGCCGGAATGTTGGATTCCGGCAAGAACGTTAATTTTTCCAAAGGAAAAAAGAAACTATTCTTGACCGTCGTTGCCTCAGTCCTACTCGTTGCAGCAGTTATCGGAGTAGTTGTCGGTGTAAAATTCCGTTCAAATAACTCCGACGACCACGCAGACATACAGGCCATTACCTCTGCAGCTCATGCCATTGTAAAATCTGCGTGTGAAAACACCCTGCACCCCGAATTATGTTACTCTACAATTGCAAGTGTTTCAGATTTCTCCAAAAAAGTAACTAGCCAAAAAGACGTGATTGAGTTGTCCTTGAATATCACCTGCAGGGCCGTCCAACACAACTTTTTTAAGGTCGAAAAACTCATCAAAACAAGAAAAGGTCTAAAGCCACGAGAAAAGGTTGCGTTGCACGATTGCCTTGAGACAATAGACGAGACTCTCGACGAACTCCACACGGCCATAAAAGACCTCGAGCTATATCCCAACAAAAAGTCTCTGAAAGCCCACGCAGATGACCTTAAAACTCTAATAAGTTCAGCAATTACTAACCAAGAGACTTGCCTTGACGGTTTCTCTCACGACGACGCCGATAAAAAGGTTCGCAAAGCTTTGCTGAAAGGTCAAAAGCACGTAGAAAAAATGTGCAGTAATGCTTTAGCTATGATTTGTAATATGACCGATACAGACATTGCTAATGAGCAGAAGTTGAAGGGTACCACTACTAACAGGAAGTTGAGAGAGGACAATAGTGAGTGGCCGGAGTGGTTGTCTGCTGGGGACAGAAGATTGCTGCAGTCCTCGACGGTGAGGCCCGACGTGGTTGTGGCGGCGGACGGAAGCGGAAATTTCAAGACGGTGTCGGAGGCGGTAGCAAAAGCACCAGAGAAGAGCAGCAAGAGGTATGTAATAAGGATAAAAGCTGGTGTTTACAGGGAGAATGTAGATGTGCCGAAGAAGAAGACAAATATTATGTTTATGGGAGATGGAAGAAGCAATACTATTATTACAGGAAGTAGAAATGTGAAAGATGGTAGCACTACTTTCCACTCCGCCACTGTTGCTGCGGTAGGTGAAAAATTCTTGGCCCGAGACATAACTTTCCAAAACACTGCAGGGGCCGCAAAACACCAAGCCGTAGCACTTCGCGTGGGGTCTGATTTGTCCGCTTTCTATAGATGTGACATTTTAGCCTATCAAGACTCTCTCTACGTTCACTCCAATCGTCAATATTTTGTTCAGTGTTTAATTGCGGGCACTGTTGATTTTATTTTCGGCAATGCTGCTGCTGTTTTACAAGACTGTGATATTCATGCCCGGCGTCCTGGTTCGGGTCAGAAGAATATGGTCACAGCCCAAGGAAGATCTGACCCGAACCAGAACACTGGAATTGTGATCCAGAAATGTAGAATTGGTGCAACGTCTGATTTGAGACCAGTTCAGAAGAGTTTCCCCACGTATCTTGGACGGCCATGGAAAGAATATTCTAGGACTGTGATTATGCAATCGTCAATTACTGATGTGATTAATTCTGCTGGATGGCATGAATGGAATGGGAATTTTGCGCTTAATACTTTGTTTTATGGCGAGTATCAAAATACTGGGGCAGGAGCTGGGACCTCAGGAAGAGTTAAATGGAAAGGGTTTAAGGTCATTACAAGTGCAACTGAGGCTCAAGCTTATACTCCTGGTAGATTCATTGCTGGAGGTAGTTGGTTGAGCTCCACTGGCTTCCCTTTTTCTCTTGGTCTCTGAAATCCTTTATTTTAAGTAGTATTTTGTTTTCAAGTTTTTGTGAAGTTTCATGAGAAATGTTTATTTTGAGCCGGAGGACACTCATG**CATGGCAATGGTGTTTGGCTGA**TGGAGAAATAACAAGATTCATCAACGAATAGGGGTTGGAAATTTTCTTTATGAACTTCTATTCGTGCTGAGGAATTTTAGTGATATAGACCTAAATATTCCCTAATGTGTGTAATTATCCTATCTAAAAGCGAAAAGCCAAAAGAATGTGGAAATAAAATAAGAGAATTTCAAAGCTCCATATA

**Tag code: Tag_23336**

PREDICTED: *Nicotiana tomentosiformis* auxin-responsive protein IAA13 (LOC104113345), transcript variant X4, mRNA

Encode proteins were deduced by BLAST search: Auxin-responsive protein IAA13 isoform X4 (*Nicotiana tomentosiformis*) NCBI Reference Sequence: XP_009621770.1

AGACATAAGACATTACTGCTGGGGACCGTTAGGAGAAAAATCATCATCCTATTCATCCTCCGCTGAAGCGCACAAAGCAGACAGGTGGTACCCCACTTCTCCCTCCTTTTTCTACCTTCTCATACACTCCCCCCCACCCTCACCTACCTTTCATTACCATTTCCTTTCTCCCCATTTGCTCTTCTTTTCTTCTTTCCCTTCTTACCCACCTGCAAAAAAATCAAGAAACCCCTCTCTGTTTTTTGATGGAAGCTACTCTTGGTCTAATGGATTTAGCTGGTGATGGTGGAGGAGGAAGTGACGGGTCAATTTCAACAGTGTCAAAGGAAGAGAATATGGTGTTGTTATCATCTGAGGCTTCTTCATCATCATACCCTGATGAGTCTGAGCTTGAATTGGGTCTTGGACTTAGTCTTGGTGCTGCTGTTTCTAAGTCTAATAAAACTGGAGCTTCAAGGGCTAAATTCTTGACGGCTAAAGATTTCCCTTCTTTGGGTTCCAATTCATTTTCTTCATCTTCCTCTTCTTCAGCTACTAAACTTAATAATAATGCCTCCTGTGGCACCAAGAGAGCTGCTGATTCTATTTCACCTCCCCGTTCTACTGTCAGTCATGTTGTTGGATGGCCTCCTGTAAGAACTTATAGGATGAATACCCTAGTTAACCAGACAAAATCACCACCCACAGAAGAATATTGCGGGACAATTGAGAAATGCAAAAGCAAAATTTTTATCACTAACGGGGGGAGCAGCAAGAGCAACAGTTTTGCCAAGGAGAAAGGGCTCATCAAGACTTCCATGTTTGTGAAGGTCAATATGGATGGAGTTGGAATTGGAAGGAAGGTAGATCTGAATGCTCATAGTAGCTATGAGAACTTGGAACGGACTTTAGATAGGATGTTCTTAAAACCCAACACAGCGGTTTGTGCAAGATCGTCAAATGCACAAGAGCTAGGTGTAATGTCAGAAACGTCATCTTCAAGATTATTAGATGGATCTACAGAATTTGTGCTAACTTATGAAGACAAAGAAGGAGACTGGATGCTTGTTGGAGATGTTCCATGGGACTCCAAGTTTCATGGAAAGAAATGGAAGACAGAGGACTAAGCCTATATAGGTTAGATAAAGAATCTACTTTCAAAGATAAAGAAGCCCCTTATTGGAAGGATGAAGTACAGAAGGAAATTATAGTGGATTATAAAATAATTTCTCAGTAACAAACTGATCATATGTGACCTTTATTGGCCTTTTATTTTTTTGTCACTTTTTTCCTGCTTTTTACCACACGGGTCATGGTGTTTAC**CCCACTCTCTGGGAGGAGA**GACCTTTTGGTAGATTACAGAAGATGCTGGTATATGTCATACACTATGCAATCTTCAGAGGAAGTTGCTTTCTGCCTTGTGAGTATGGATATGTCTACTTATATTATATTACGATGATGAGCTTGCCGCACCCTGAAACAGATGAACGCTAATGTTGTTGAAAGGAAAATTTGCAAGGTTCATACTGGGAACCTTTTTTGTTGGTTTTGATAAAAGATTTCATTGCTTCATGTTTGTATTTCAA

Additional Primers:

polyT anc: GGCCACGCGTCGACTAGTACTTTTTTTTTTTTTTTTT

26S rRNA Fw CACGGACCAAGGAGTCTGACAT

26S rRNA Rv TCCCACCAATCAGCTTCCTTAC
